# Region-Based Association Test for Familial Data under Functional Linear Models

**DOI:** 10.1371/journal.pone.0128999

**Published:** 2015-06-25

**Authors:** Gulnara R. Svishcheva, Nadezhda M. Belonogova, Tatiana I. Axenovich

**Affiliations:** 1 Institute of Cytology and Genetics, Siberian Branch of the Russian Academy of Sciences, Novosibirsk, Russia; 2 Department of Natural Sciences, Novosibirsk State University, Novosibirsk, Russia; University of North Carolina, UNITED STATES

## Abstract

Region-based association analysis is a more powerful tool for gene mapping than testing of individual genetic variants, particularly for rare genetic variants. The most powerful methods for regional mapping are based on the functional data analysis approach, which assumes that the regional genome of an individual may be considered as a continuous stochastic function that contains information about both linkage and linkage disequilibrium. Here, we extend this powerful approach, earlier applied only to independent samples, to the samples of related individuals. To this end, we additionally include a random polygene effects in functional linear model used for testing association between quantitative traits and multiple genetic variants in the region. We compare the statistical power of different methods using Genetic Analysis Workshop 17 mini-exome family data and a wide range of simulation scenarios. Our method increases the power of regional association analysis of quantitative traits compared with burden-based and kernel-based methods for the majority of the scenarios. In addition, we estimate the statistical power of our method using regions with small number of genetic variants, and show that our method retains its advantage over burden-based and kernel-based methods in this case as well. The new method is implemented as the R-function ‘famFLM’ using two types of basis functions: the B-spline and Fourier bases. We compare the properties of the new method using models that differ from each other in the type of their function basis. The models based on the Fourier basis functions have an advantage in terms of speed and power over the models that use the B-spline basis functions and those that combine B-spline and Fourier basis functions. The ‘famFLM’ function is distributed under GPLv3 license and is freely available at http://mga.bionet.nsc.ru/soft/famFLM/.

## Introduction

Despite the massive success of genome-wide association studies (GWAS), a significant part of the heritability of quantitative traits remains unexplained. Studying the role of rare genetic variants in the etiology of complex traits may solve the problem of missing heritability [1]. Rapid progress in whole-exome and whole-genome sequencing technologies provides new opportunities for detecting rare variants that control complex traits. However, the statistical methods based on single genetic variant association tests commonly used in GWAS are underpowered for rare variants due to their uncommon occurrence. The statistical power of association analysis is therefore expected to increase when genetic variants in a region of interest are tested simultaneously, instead of separately [2, 3].

Several approaches have been suggested for region-based association analysis of rare variants. The first, simplest of them, employs burden tests based on collapsing rare variants within a region of interest [2, 4–7]. For this approach, a set of rare variants in the region is reduced to a single genetic variable that is then tested for association through conventional GWAS methods [2, 4–6]. The computational complexity of that regional association analysis is similar to the complexity of GWAS. The fast algorithms have been developed for GWAS even for structured samples (e.g., [8–11]). However, the collapsing approach assumes that the majority of rare variants are causal and that their effects are unidirectional. The power of association analysis decreases if these assumptions do not hold [12].

The second approach employs kernel machine regression for regional association analysis [13–17]. This method compares the average similarity of genetic variants’ set in the analyzed region for each pair of individuals with pairwise phenotypic similarity. The pairwise genetic similarity is measured using a kernel function, that reduces information on multiple genetic variants for a pair of individuals into a single scalar factor. Regional association analysis typically uses linear kernel functions to describe the genetic similarity.

In contrast to collapsing-based methods, kernel-based methods are more resilient to the opposite direction of causal variant effects and the limited proportion of causal variants [17–19]. Several software programs have been developed to carry out kernel-based association tests using both independent [17, 19, 20] and family-based samples [21–25].

Both burden-based and kernel-based approaches have been combined in optimal unified test implemented in SKAT-O [19] and MONSTER [26] programs, which are adapted for independent and structured samples, respectively. This optimized test has a higher power than the kernel-based test in case when causal variants are unidirectional [19].

However, burden-based and linear kernel-based approaches, as well as their optimal combination, only partly utilize information about linkage and linkage disequilibrium (LD) of genetic variants. In burden-based methods, the LD pattern may be missing or reduced after collapsing rare genetic variants into a single variable. Linear kernel-based methods utilize only pairwise LD in their kernel matrix. They do not model higher-order LD among the genetic variants [27].

The third approach, based on a functional data analysis (FDA), allows for better utilization of information on LD and linkage in regional analysis of association between rare variants and both quantitative [27, 28] and dichotomous [29–31] traits. This approach takes into account not only a set of genotypes of genetic variants within the region of interest, but also the physical locations of these variants (i.e., their order and the distances between them). An individual’s multiple densely located genetic variants can be considered as a continuum of sequence data rather than discrete observations, and therefore can be treated in the FDA as a realization of a stochastic process [32–34]. Thus, the genome of an individual can be regarded as a stochastic function that contains both linkage and LD information.

The FDA-based method of regional association analysis of quantitative traits has been applied to the independent samples [27, 28]. Simulation analysis demonstrates that FDA test using functional linear models has a higher power than sequence kernel association test for most scenarios. However, the FDA approach to regional association analysis has not yet been applied to structured samples and it remains unclear whether its statistical properties change when the number of variants in the region is small. The situation with the small number of variants is important for the exome studies. There, the analyzed variants are often limited to non-synonymous substitutions, missense, stop-gained, and stop-lost mutations, the number of which is small in some genes.

In this paper, we extend the FDA approach to samples with (sub)structure and implement the method in a computer program. We compare the power of our new method to the burden-based, kernel-based methods, and their optimized combination using Genetic Analysis Workshop 17 mini-exome family data [35] and a wide range of simulation scenarios. In addition, we consider the case when the regions have a small number of genetic variants.

## Materials and Methods

### Model

Consider a genomic region containing *m* genetic variants with known physical locations *t*_*i*_(*i* = 1,…,*m*). We order these locations, 0 ≤ *t*_1_ <…< *t*_*m*_ = *T*, and scale the region size from [*t*_1_,*T*] to [0,1].

For a family-based sample of *n* individuals, let *y* denote an (*n*×1) vector of known trait values, *X* denote an (*n*×*c*) matrix of the *c* covariates such as age and sex, and *G* denote an (*n*×*m*) matrix of genotypes of *m* variants. Here, *G*_*ij*_ is equal to the number of minor alleles of the *i*-th individual at the *j*-th variant with the location *t*_*j*_.

The traditional linear mixed model used to analyze a family-based sample is

y=Xα+Gβ+h+e. (1)

Here *α* is a (*c*×1) fixed vector of regression coefficients measuring the effects of the *c* covariates; *β* is an (*m*×1) fixed vector of regression coefficients measuring the effects of the *m* genetic variants; *h* is an (*n*×1) random vector of polygene effect values distributed as $N(0,\sigma_{h}^{2}R)$, and *e* is an (*n*×1) vector of random errors distributed as $N(0,\sigma_{e}^{2}I)$, where $\sigma_{h}^{2}$ and $\sigma_{e}^{2}$ are respective variance components, and *R* and *I* are (*n*×*n*) relationship and identity matrices, respectively. Model (1) assumes that the phenotypes *y* follow a multivariate normal distribution with a mean vector *E*(*y*) = *Xα*+*Gβ* and a covariance matrix $\Omega =\sigma_{h}^{2}R+\sigma_{e}^{2}I$.

For each related individual, we interpret discrete genotypic values of ordered variants in a region of interest as continuous data by using functional linear analysis techniques. Such an interpretation is possible because of the high density of genetic variants, whose genotypes are continuous rather than discrete observations. These techniques have been applied to region-based association analysis for unrelated samples in [27].

For family-based samples, we introduce a functional linear mixed model as

$$y=X\alpha +\int_{0}^{1}\tilde{G}\left(t\right)\tilde{\beta }\left(t\right)dt+h+e.$$ (2)

Here, $\tilde{G}\left(t\right)={\left({\tilde{G}}_{1}\left(t\right),\ldots ,{\tilde{G}}_{n}\left(t\right)\right)}^{T}$ denotes an (*n*×1) unknown vector of genetic variant functions (GVFs), and $\tilde{\beta }\left(t\right)$ denotes an unknown continuous beta-smoothing function (BSF) of *t* in [0,1]. In contrast to the functional linear model used in the analysis of independent samples, the proposed model additionally includes a random polygenic effect *h*.

Our goal is to find a functional vector $\tilde{G}\left(t\right)$ such that its discrete realization ${\tilde{G}}_{i}\left(t_{j}\right)$ becomes as close as possible to real *G*_*ij*_,*i* = 1,…,*m*, and to define a function $\tilde{\beta }\left(t\right)$ to smooth the regression coefficients. We select a system of basis functions $\left\{\phi_{1}\left(t\right),\ldots ,\phi_{K_{G}}\left(t\right)\right\}$, whose linear combinations allow us to approximate each of $\tilde{G}\left(t\right)$. The values of these basis functions in all positions *t*_*j*_(*j* = 1,…,*m*) are represented as an (*m*×*K*_*G*_) matrix *Φ*, for which the closest orthogonal matrix is *Φ*(*Φ* ^*T*^ *Φ*)^−1^. The GVFs may thus be written for *t* ∈ [0,1] as in [32], Chapter 4:

$$\tilde{G}\left(t\right)=G\Phi {\left(\Phi^{T}\Phi \right)}^{-1}\phi \left(t\right),$$ (3)

where $\phi \left(t\right)={\left(\phi_{1}\left(t\right),\ldots ,\phi_{K_{G}}\left(t\right)\right)}^{T}$. Note that to perform matrix inversion in expression (3), the number of basis functions must be no more than the number of genetic variants in the region (i.e., *K*_*G*_ ≤ *m*).

Similarly, using other (or the same) system of *K*_*β*_ basis functions $\left\{\psi_{1}\left(t\right),\ldots ,\psi_{K_{\beta }}\left(t\right)\right\}$ (see details in S1 Note), we can estimate the BSF in Model (2) as

$$\tilde{\beta }\left(t\right)=\psi^{T}\left(t\right)\beta_{F},$$ (4)

where $\beta_{F}={\left(\beta_{F_{1}},\ldots ,\beta_{F_{K_{\beta }}}\right)}^{T}$ is a (*K*_*β*_×1) vector of model regression coefficients and $\psi \left(t\right)={\left(\psi_{1}\left(t\right),\ldots ,\psi_{K_{\beta }}\left(t\right)\right)}^{T}$.

Substituting estimates (3) and (4) into Model (2), we obtain a functional linear regression model including fixed effects (*α* and *β*) and random effects (*h* and *e*):

$$y=X\alpha +GW\;\beta_{F}+h+e,$$ (5)

where $W=\Phi {\left(\Phi^{T}\Phi \right)}^{-1}\left[\int_{0}^{1}\phi \left(t\right)\psi^{T}\left(t\right)dt\right]$. From the way *Φ* and the integral are defined, we see that *W* depends only on the given basis functions and positions of genetic variants in the region.

The matrix *W* can be represented as a product of two matrices *W*_1_ and *W*_2_, where *W*_1_ = *Φ*(*Φ* ^*T*^ *Φ*)^−1^ of dimension (*m*×*K*_*G*_), and $W_{2}=\int_{0}^{1}\phi \left(t\right)\psi^{T}\left(t\right)dt$ of dimension (*K*_*G*_×*K*_*β*_). Unlike the matrix *W*_1_, the matrix *W*_2_ is independent of the real data being analyzed. It is defined only by a selected set of basis functions and is the same for all regions if the number of basis functions is fixed. However, to uniquely and correctly estimate the parameters *β*_*F*_ in Model (5), the restriction *m* ≥ *K*_*G*_ ≥ *K*_*β*_ must be introduced; otherwise the matrix *W* used for *β*_*F*_ estimation is not invertible (see details in S2 Note).

Note that *W* is an (*m*×*K*_*β*_) transition matrix between the vector *β* in Model (1) and the new vector *β*_*F*_ in Model (5). The number of elements of the new vector *β*_*F*_ is at most the one of the vector *β*, because *K*_*β*_ ≤ *m*. This allows us to decrease the number of parameters describing the model.

In addition to Model (5), we construct a simplified functional linear model, which directly uses the genotype data. In this case, only beta smoothing function *β*(*t*) is estimated, using a system of *K*_*β*_ basis functions:

$$y=X\alpha +G\Psi \beta_{F}+h+e.$$ (6)

In the formula (6), the *m*×*K*_*β*_ matrix *Ψ* is constructed analogously to the *m*×*K*_*G*_ matrix *Φ* in Model (5), which depends on the selected function basis and the genetic variant positions; that is, an element *Ψ*_*ij*_ of the matrix *Ψ* is a value of the *i*-th basis function in the *j*-th position. Therefore, we obtain Model (6) from Model (1), performing the single replacement *β = Ψβ*_*F*_.

### Statistical test

In a framework of the functional linear Model (5), we treat the fixed effects *β*_*F*_ as unknown constant parameters. To check the associations between the genomic region and the quantitative trait, we test the null hypothesis *H*_0_: *β*_*F*_ = 0 against *H*_1_: *β*_*F*_ ≠ 0. As in the case of independent samples, the null hypothesis *H*_0_ may be tested using *F*-statistic with degrees of freedom *K*_*β*_ and *n* − *K*_*β*_ − 1, see [36]. However, for samples of related individuals an *F*-statistic is defined as:

$$F=\frac{\left(RSS_{0}-RSS_{1}\right)/K_{\beta }}{RSS_{1}/\left(n-K_{\beta }-1\right)},$$

where *RSS*_0_ = (*y* − *Xα*)^*T*^Ω^−1^(*y* − *Xα*) and *RSS*_1_ = (*y* – *Xα* – *GW β*_*F*_)^*T*^Ω^−1^(*y* − *Xα* – *GW β*_*F*_) are the weighted sums of the correlated squared residuals under the null and alternative models, respectively.

To calculate *F-*statistic for a sample of related individuals, the maximum likelihood estimates of covariance matrix Ω and the vector of regression coefficients *α* are obtained under the null hypothesis. The vector of regression coefficients *β*_*F*_ is estimated under the alternative hypothesis using the obtained values of Ω and *α* as

$$\beta_{F}={\left(W^{T}G^{T}\Omega^{-1}GW\right)}^{-1}W^{T}G^{T}\Omega^{-1}\left(y-X\alpha \right).$$

For independent samples, this expression does not include covariance matrix Ω.

The likelihood ratio test (*LRT*) distributed asymptotically as *χ* ^2^ with *K*_*β*_ degrees of freedom and the *χ* ^2^-distributed score test are used as alternative tests to compare hypotheses *H*_0_ and *H*_1_.

### Implementation

We implemented the new method into the R-function ‘famFLM’ using two common types of basis functions: the B-spline and Fourier bases (see details in S1 Note). The function ‘famFLM’ allows the use of both GVF and BSF, or BSF only. The type and number of basis functions can thus be set by the user. The software provides: covariates and dominance, three types of test statistic (*F*, *χ* ^2^, and *LRT*), sequential or parallel calculation, and running time estimation. Any type of relationship matrix (genome- or pedigree-based) can be used. The ‘famFLM’ function is distributed under GPLv3 license and is freely available at http://mga.bionet.nsc.ru/soft/famFLM/.

### Simulations

We used genotypes of the Genetic Analysis Workshop 17 (GAW17 [35]) family-based sample that consists of 697 individuals in eight families. This data set includes only 200 repeats of simulated traits, which are not sufficient to estimate the type I error and the power of different methods. Therefore, we simulated additional replicas of a quantitative trait with *h* ^2^ = 0.29 under *H*_0_ (as in Q2 quantitative trait of GAW17).

To estimate the type I error, we analyzed 12,636 genetic variants in 1,702 gene regions that contain more than one polymorphic exome genetic variant of the GAW17 data set. We analyzed 10^5^ replicas (1.7×10^8^ regions) to obtain the type I error estimates down to the significance level of 2.5×10^−6^.

For power estimation, we selected one region that contains 50 polymorphic genetic variants (*MAF*s ranged from 0.1% to 34.7% with median of 1%). The following scenarios were considered for simulations: 1) proportion of causal variants in the regions 0.05, 0.1, or 0.2; 2) proportion of effects that have the same direction 0.5, 0.8, or 1; 3) for each causal variant, the effect size |*β*| = log(*c*)|log_10_(*MAF*)| / 2 as in [27], with constant *c* being 2, 3, 5, or 7.

To explore the power of different methods for regions with small number of genetic variants, we simulated effects only for rare genetic variants within the region (36 rare variants with *MAF* ≤ 0.03, median *MAF* after excluding common variants: 0.6%). Subsequently, we analyzed all rare variants and rare variants after excluding 50% or 80% non-causal variants.

We analyzed the association between the quantitative traits and the genotypes of SNPs in the region using *F*-statistics for testing fixed effects in the mixed model. For each scenario, 1000 replicas were analyzed and the power was estimated as a proportion of *P* values that are less than 2.5×10^−6^.

### Comparison methods

We tested the statistical properties of the new method as compared with burden-based, kernel-based, and optimized kernel-based methods. We used the ‘famFLM’ function for the new FDA-based method. We used cubic B-spline and Fourier bases that contain 15 and 25 functions, respectively. Such values were recommended by Fan et al. [27] and tested on our data (S1 Note). If the fixed number of basis functions exceeded the number of genetic variants in a region of interest, the number of basis functions was automatically reduced to the number of genetic variants. To describe the GVFs and the BSF in Model (5), we considered all combinations of function bases: Fourier basis for both the GVF and the BSF (F-F), B-spline basis for both the GVF and the BSF (B-B), Fourier basis for the GVF and B-spline basis for the BSF (F-B), and finally, B-spline basis for the GVF and Fourier basis for the BSF (B-F). Furthermore, we considered cases in which only the BSF was described, (0-B and 0-F, in case of B-spline and Fourier bases, respectively) (Model (6)).

We used the MONSTER package for the burden-based, kernel-based, and optimized kernel-based methods [26].

### Running time

The running time of a mini-exome regional analysis was estimated on a single processor of a computer server that was equipped with 192 GB memory and two Intel Xeon E5-2650 v2 eight core 2.60 GHz processors, CentOS 6.5 Linux 2.6.32–431.29.2.el6.x86_64. We used the same data as in the type I error estimation to compare the running times of a whole mini-exome analysis using different models. The mentioned running time does not take into consideration the null model estimation step. This step is the same for all compared models, can be performed once, and took 1.56 seconds in the GAW17 family sample. To explore how the running time increases with the sample size, we used the regions with fixed *m*. To obtain samples of different size, we used a subset of 500 related individuals in 7 pedigrees from the GAW17 family sample and doubled it to generate sample sizes of 1,000, 2,000 and 4,000 individuals, respectively (as in [25]).

## Results

We estimated the empirical type I error rates for six significance levels down to 2.5×10^−6^ (Table 1). For all models that differed from each other in type of their function basis used to describe GVFs and BSF, the empirical type I error rates were close to the nominal *α* values. For one model, 0-F, we estimated the type I error rates at lower levels and found a good correspondence to the declared values (0.92×10^−7^ for *α* = 1×10^−7^ and 4.47×10^−8^ for *α* = 5×10^−8^, based on 1.7×10^9^ regional *P* values). The proposed test controls type I error rate correctly over all significance levels. Therefore, it can be used in both candidate gene and exome-wide studies (the model 0-F even in genome-wide studies).

**Table 1:** Simulation results of type I error rates of six famFLM tests.

***α***	Basis of *β*(*t*):	B-spline			Fourier		
	Basis of GVF:	B-spline	No basis	Fourier	B-spline	No basis	Fourier
**0.05**		0.04563	0.05006	0.05012	0.04960	0.04717	0.05008
**0.01**		0.00897	0.00991	0.00996	0.00982	0.00929	0.00993
**1×10** ^**−3**^		8.74×10^−4^	9.70×10^−4^	9.74×10^−4^	9.62×10^−4^	9.04×10^−4^	9.73×10^−4^
**1×10** ^**−4**^		8.50×10^−5^	9.41×10^−5^	9.50×10^−5^	9.38×10^−5^	8.76×10^−5^	9.63×10^−5^
**1×10** ^**−5**^		8.63×10^−6^	9.10×10^−6^	9.20×10^−6^	9.48×10^−6^	8.53×10^−6^	9.49×10^−6^
**2.5×10** ^**−6**^		2.00×10^−6^	2.15×10^−6^	2.19×10^−6^	2.22×10^−6^	2.07×10^−6^	2.38×10^−6^

**Fig 1:**
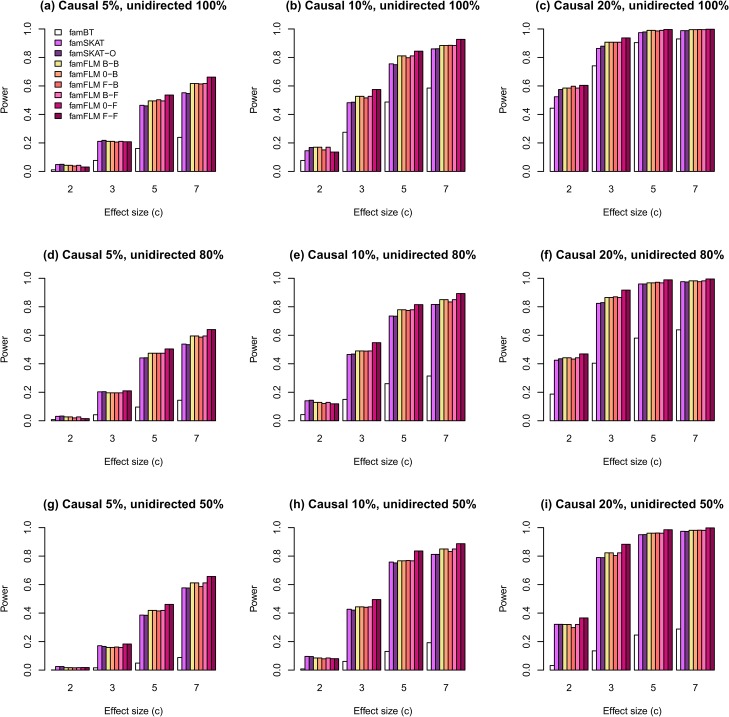
The statistical power of regional association analysis on the familial data when all (rare and common) variants were used in simulations for random selection of causal variants and in analysis. Compared methods are the burden-based (famBT), kernel-based (famSKAT), optimized kernel-based (famSKAT-O), and the new FDA-based (famFLM) methods. For famFLM, six functional models were tested: B-spline basis for both the GVF and the BSF (B-B), only the BSF described via B-spline basis (0-B), Fourier basis for the GVF and B-spline basis for the BSF (F-B), B-spline basis for the GVF and Fourier basis for the BSF (B-F), only the BSF described via Fourier basis (0-F), Fourier basis for both the GVF and the BSF (F-F).

**Fig 2:**
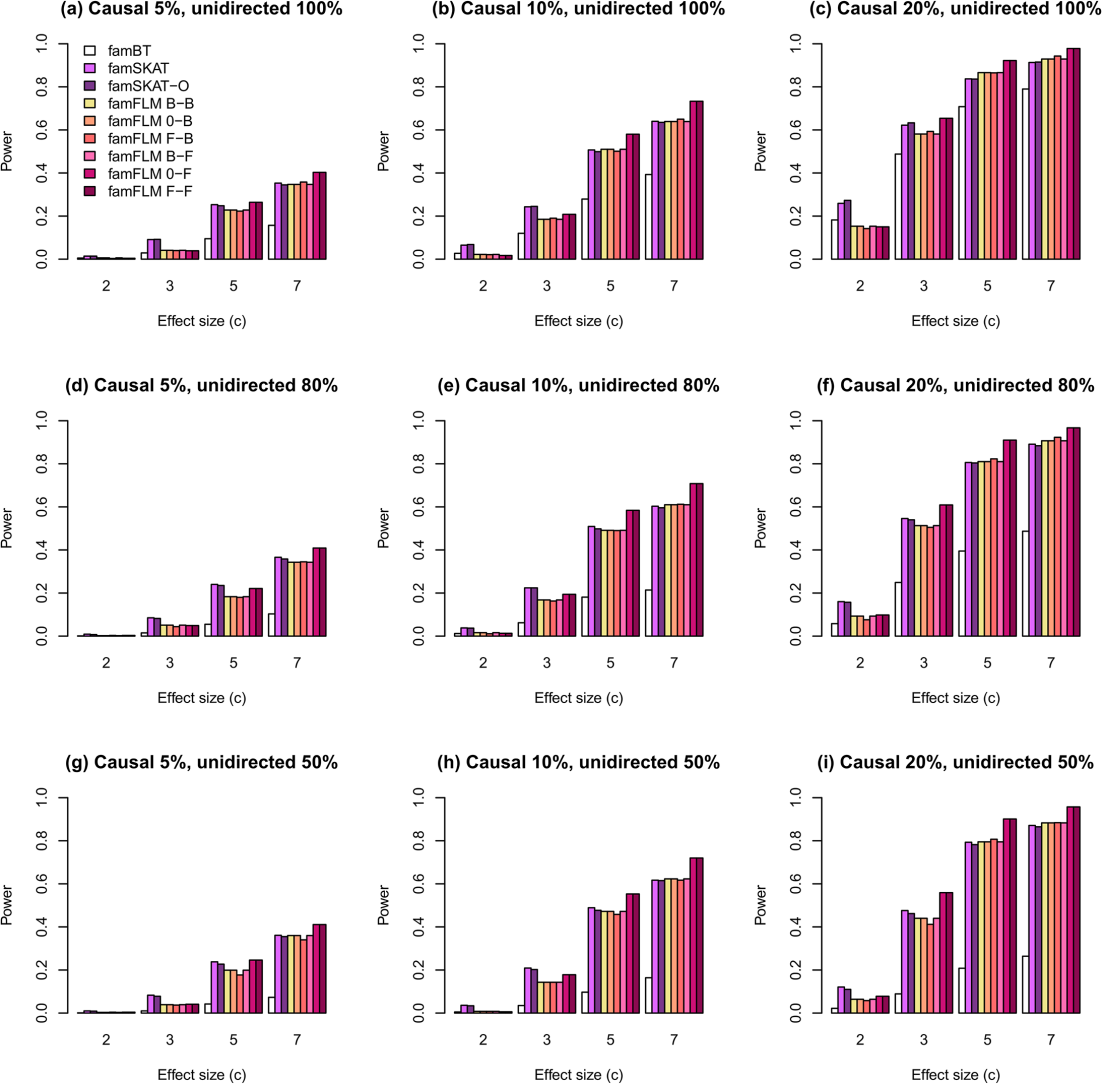
The statistical power of regional association analysis on the familial data when only rare variants were used in simulations for random selection of causal variants and in analysis. The notations of the methods are the same as in Fig 1.

**Fig 3:**
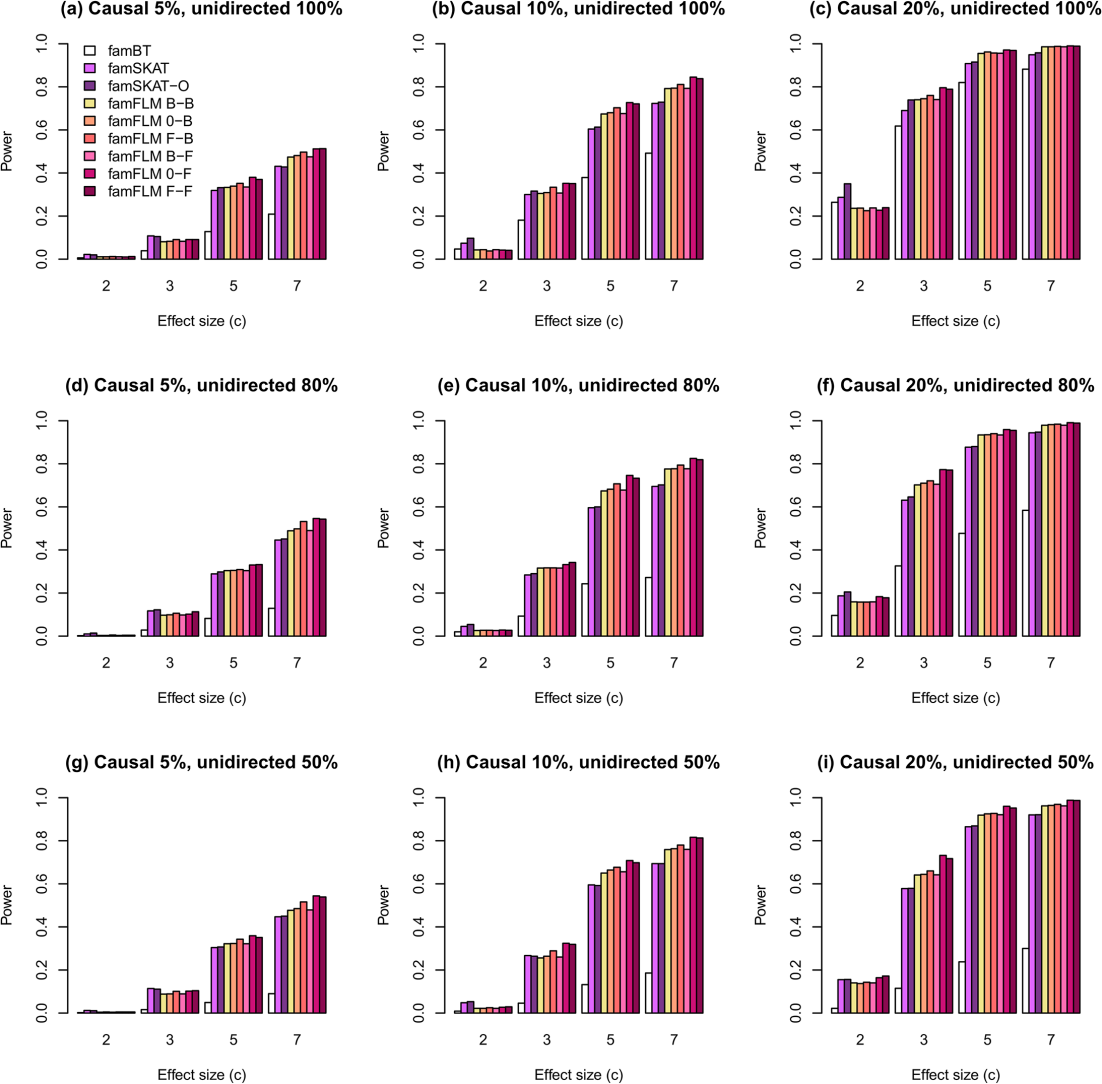
The statistical power of regional association analysis on the familial data when only rare variants were used in simulations for random selection of causal variants and 50% of non-causal variants were excluded from the analysis. The notations of the methods are the same as in Fig 1.

**Fig 4:**
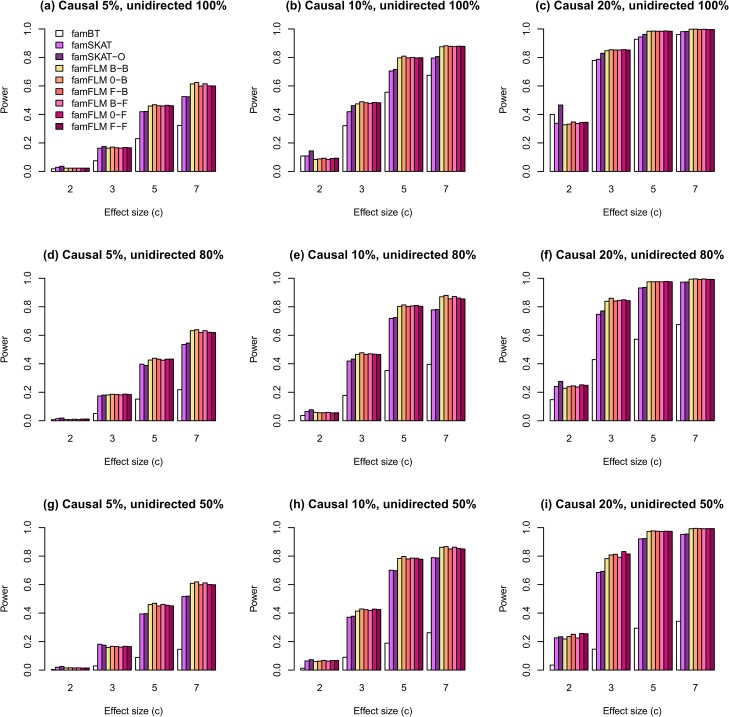
The statistical power of regional association analysis on the familial data when only rare variants were used in simulations for random selection of causal variants and 80% of non-causal variants were excluded from the analysis. The notations of the methods are the same as in Fig 1.

Estimates of statistical power for different scenarios are shown in Figs 1–4. The first set of scenarios employs both rare and common variants within the region for random selection of causal variants and for regional association analysis. As shown in Fig 1, the new method demonstrates the highest power for most scenarios. The proportion of unidirectional causal variant effects does not affect the power of any methods, except for the burden-based one. The burden-based method has the lowest power in comparison with kernel-based and functional tests, particularly for scenarios with different directions of causal variant effects (Fig 1). A similar pattern was observed for the second set of scenarios in which only rare variants were used for random selection of causal variants and for regional association analysis (Fig 2). However, the power estimates for rare variants were lower than the estimates for both common and rare variants. The results obtained for rare and both rare and common variants are similar to the results obtained earlier for independent samples described in [27]. Figs 3 and 4 show the results for the third and the fourth sets of scenarios where 50% and 80% of non-causal variants were excluded from the analysis, respectively. The median numbers of genetic variants in a region were 19, 20, and 22 after excluding 50% of non-causal variants, for scenarios with proportions of causal variants equal to 0.05, 0.1, and 0.2, respectively. These numbers were 9, 10, and 13 for the corresponding scenarios with 80% of excluded non-causal variants. The power for all tested models increased with increasing the proportion of variants removed. The new method demonstrates the highest statistical power for most scenarios not only for the first and second, but also for the third and fourth sets.

We compared the power of the new method for six functional linear models that differ from each another in type of their function basis used to describe both the GVF and the BSF or the BSF only. The method under 0-F and F-F models demonstrated the highest power for most scenarios (Figs 1 and 2). However, when we excluded a part of non-causal variants from the analysis, the difference between all six models decreased (Fig 3) and almost disappeared when 80% of non-causal variants were excluded (Fig 4). The explanation for this phenomenon is given in S2 Note.

All the above features of the power of the new method were confirmed for a significance level of *α* = 1×10^−4^ (see S1–S4 Figs).

Table 2 gives the running time of a mini-exome association analysis under different models. Models that use the FDA approach for the BSF only (0-B and 0-F) proved to have the fastest running time. Among the other models, the minimum and maximum running times were observed in the models using Fourier (F-F) and B-spline (B-B) bases, respectively. Models using both Fourier and B-spline bases (B-F and F-B) demonstrated intermediate running time. In general, the models that use Fourier basis were faster than corresponding ones that use B-spline basis. All running times, however, differed from each other by at most a factor of two. Moreover, when we analyzed how the running time depends on the sample size using only regions with fixed *m* > *K*_*G*_ ≥ *K*_*β*_, all six models demonstrated very similar results (see S3 Note). This means that the differences in running times between models shown in Table 2 are due to recalculations of the matrix *W*_2_ for restricted number of basis functions and regions with small number of genetic variants (*m* < *K*_*G*_ and/or *m* < *K*_*β*_). The matrix *W*_2_ is not involved in the models 0-F and 0-B (see formula (6)) and therefore these models showed minimal running time (Table 2). The number of *W*_2_ recalculations for the model F-F is larger than for the model B-B because the former is constructed on 25 basis functions (and *W*_2_ is recalculated for the regions with *m* < 25), while the latter uses only 15 functions and *W*_2_ is recalculated only for the regions with *m* < 15. Nevertheless, the running time for the model F-F is smaller than for B-B. This result may be explained by the specific details of B-spline and Fourier basis function computations (see S1 Note).

**Table 2:** The running time for six variants of famFLM in performing the regional-based analysis of a mini-exome.

Basis of *β*(*t*)	Basis of GVF	Running time, s
**B-spline**	**B-spline**	58.018
	**No basis**	34.801
	**Fourier**	51.552
**Fourier**	**B-spline**	53.721
	**No basis**	33.502
	**Fourier**	44.373

For the regions with fixed *m* > *K*_*G*_ ≥ *K*_*β*_, running time increases quadratically with increasing the sample size and linearly with increasing the number of genetic variants in the region (see details in S3 Note).

## Discussion

The FDA-based approach [27, 30] is currently most effective in regional association analysis of unrelated samples. In the present work, we extended it to samples of related individuals. The new method has a higher power than both the burden-based and the kernel-based methods. It introduces a new powerful tool for the analysis of rare variants in samples of related individuals from families or isolated populations, and in large human samples, where some degree of relatedness or population stratification is unavoidable.

We compared the powers of the new method using six models: F-F, F-B, B-B, B-F, 0-B, and 0-F and found two main features. Firstly, we have not seen a difference between models that use the FDA approach for the BSF only and for both the GVF and the BSF. The powers of the method using 0-B and B-B models were very close, as well as for 0-F and F-F models. The same results were obtained on unrelated samples by Fan et al. [27]. To explain this finding we analytically studied the models when *K*_*G*_ = *K*_*β*_, and demonstrated that models using the FDA approach for both the GVF and the BSF, and for the BSF only are equivalent (see S2 Note). For the F-B model, the condition *K*_*G*_ = *K*_*β*_ is not satisfied (*K*_*G*_ > *K*_*β*_) and consequently this model is not equivalent to the 0-B model (see S2 Note). However, we demonstrated that the powers for F-B and 0-B models are very close. This indicates that the FDA-based test for regional association analysis of quantitative traits does not strongly depend on whether the genotype data are smoothed or not. For such traits, the essence of FDA approach is the smoothing of the genotype effects on the trait but not the smoothing the genotypes themselves. For binary traits, in contrast, the FDA approach is used for the GVF only and a quality of genotype data smoothing is very important for the power of association test [31].

Secondly, for most scenarios, our method showed the highest power under the models using Fourier bases (0-F and F-F). The same results were obtained on unrelated samples by Fan et al. [27]. We can explain these results by the linkage disequilibrium, through which we obtain the association signals not only in the points of causal variants, but also in the neighboring areas. In other words, the signal of association does not look as a sharp peak; the signal is smoothed and spread over the site of the genome around the causal variant. Usually, the causal variants are separated by a large number of non-causal variants and extended smoothed signals in the positive or negative areas can be described by a smoothing function without strong local features. A Fourier series is especially useful for such functions, while B-spline basis is appropriate for data where discontinuities in the function itself or in low order derivatives are known or suspected (see S1 Note and [32], Chapter 3).

To use the FDA approach, we should select not only the types of basis functions, but also the number of these functions. If this number is small, we may miss some important aspects of the smoothing function that we are trying to estimate. It is obvious that the larger the number, the better the fit to the data. However, in this case we risk also fitting noise or variation that we wish to ignore. It is generally unclear how to choose the optimal number of basis functions. Usually two algorithms are used [32], Chapter 4. One of them, the stepwise variable selection algorithm, adds basis functions one after another, tests at each step whether the added function significantly improves the fit, and also checks that the functions already added continue to play a significant role. Other variable-pruning algorithm is used for high-dimensional models; it removes basis functions one after another, starting with a generous amount of basis function. However, the existing algorithms are computationally intensive; they are especially time-consuming for analysis of associations between multiple genome regions within exome and quantitative traits where sample size is usually large. Fan et al. [27] demonstrated that the fixed number of basis function can be used for association analysis of quantitative traits in unrelated samples. We confirmed this conclusion using other data and a sample of relatives (S1 Note). The possibility to use fixed number of basis functions has a large practical importance, because it decreases the running time manifold.

The FDA approach suggests that multiple genetic variants in the region are genotyped, therefore allowing us to consider the individual’s genotype of a particular genomic region as a continuous function of variants’ physical locations in the region rather than a set of discrete values. However, a considerable proportion of variants that come from re-sequences are not polymorphic in the restricted sample of individuals, and some polymorphic variants in exome are synonymous. Often, only non-synonymous substitutions, missense, stop-gained, and stop-lost mutations are included in regional association analyses. The number of such variants in the region can be rather small. This presents the question of whether the new method continues to perform better than other existing methods if the number of variants in the region is small. To test our method in such a situation, we excluded a part of non-causal variants from the analysis. In addition, we adapted our method to the analysis of regions with small number *m* of genetic variants, specifically when *m* < *K*, where *K* is the number of basis functions. Given small *m*, we automatically limited *K* in our algorithm and its implementation in the ‘famFLM’ function. In the case when *K* = *m*, the functional linear regression model (5) analytically reduces to the traditional linear mixed model (1) (see S2 Note).

The model of multiple linear regression includes genotypes of all genetic variants as predictors. The number of degrees of freedom for this model is automatically fixed as *m* under the condition of linear independence of the genotype vectors of the variants. However, usually not all variants in the region are causal. In this case, fixing the number of degrees of freedom larger than the number of causal variants can reduce the power. The fewer the number of non causal variants in the region, the smaller is the power loss. In our ‘famFLM’, the multiple linear regression is used only for small *m* ≤ *K*, and this small size can be due to exclusion of synonymous substitutions. We demonstrated that in this case our method has a clear advantage in comparison with burden-based and kernel based methods. It was shown that the increase of the proportion of causal variants similarly increases the association power for burden-based and kernel-based methods [2]. In this study, we demonstrated that our method also has this property, and that such a property remains when the number of variants in the region is small.

In the present study, we restricted our analysis by the functional linear models for *F-*testing the fixed effects of genetic variants, although these models can also be used for variance component analysis by means of the functional kernel score test (FKST). In this case the FDA-approach is applied to the kernel matrix which describes the similarity of genetic variants’ set in the analyzed region for each pair of individuals [27]. The problem is that the type I errors of FKST do not correspond to the nominal levels for *α* < 0.01 and this test has less power than the *F-*test [27]. Even though it was demonstrated that the FKST-based method has a high power when sample size is small and the region includes a single causal variant [27], its statistical properties should be studied in more details before its practical application.

Introducing the FDA technique in regional association analysis allows for a more complete use of information on the genetic structure of the analyzed genome regions. All existing methods operate only with a set of genetic variants in the region, whereas the new method also utilizes information about the order of genetic variants and the distances between the variants. This new feature allows us to include pairwise linkage disequilibrium (as existing methods do), as well as information on linkage and higher-order linkage disequilibrium in the model.

Previously, the new FDA-based approach was developed only for samples of independent individuals. The present study extends this approach to samples of genetically related individuals. The new method provides a powerful tool in identification of rare variants involved in the control of quantitative traits.

## Supporting Information

S1 Fig The statistical power of regional association analysis on the familial data when all (rare and common) variants were used in simulations for random selection of causal variants and in analysis (*α* = 1×10^−4^). The notations of the methods are the same as in Fig 1.
(PDF)

S2 Fig The statistical power of regional association analysis on the familial data when only rare variants were used in simulations for random selection of causal variants and in analysis (*α* = 1×10^−4^). The notations of the methods are the same as in Fig 1.
(PDF)

S3 Fig The statistical power of regional association analysis on the familial data when only rare variants were used in simulations for random selection of causal variants and 50% of non-causal variants were excluded from the analysis (*α* = 1×10^−4^). The notations of the methods are the same as in Fig 1.
(PDF)

S4 Fig The statistical power of regional association analysis on the familial data when only rare variants were used in simulations for random selection of causal variants and 80% of non-causal variants were excluded from the analysis (*α* = 1×10^−4^). The notations of the methods are the same as in Fig 1.
(PDF)

S1 Note Basis functions. (PDF)

S2 Note FLM-based association analysis under different ratios between *m*, *K*_*G*_ and *K*_*β*_. (PDF)

S3 Note Effect of the sample size and the number of genetic variants on the running time of famFLM test. (PDF)

## References

[1] Bansal V, Libiger O, Torkamani A, Schork NJ. Statistical analysis strategies for association studies involving rare variants. Nature reviews Genetics. 2010;11(11):773–85. 10.1038/nrg2867 20940738 PMC3743540

[2] Li B, Leal SM. Methods for detecting associations with rare variants for common diseases: application to analysis of sequence data. Am J Hum Genet. 2008;83(3):311–21. 10.1016/j.ajhg.2008.06.024 18691683 PMC2842185

[3] Eichler EE, Flint J, Gibson G, Kong A, Leal SM, Moore JH, et al Missing heritability and strategies for finding the underlying causes of complex disease. Nature reviews Genetics. 2010;11(6):446–50. 10.1038/nrg2809 20479774 PMC2942068

[4] Madsen BE, Browning SR. A groupwise association test for rare mutations using a weighted sum statistic. PLoS Genet. 2009;5(2):e1000384 10.1371/journal.pgen.1000384 19214210 PMC2633048

[5] Morris AP, Zeggini E. An evaluation of statistical approaches to rare variant analysis in genetic association studies. Genet Epidemiol. 2010;34(2):188–93. 10.1002/gepi.20450 19810025 PMC2962811

[6] Price AL, Kryukov GV, de Bakker PI, Purcell SM, Staples J, Wei LJ, et al Pooled association tests for rare variants in exon-resequencing studies. Am J Hum Genet. 2010;86(6):832–8. 10.1016/j.ajhg.2010.04.005 20471002 PMC3032073

[7] Han F, Pan W. A data-adaptive sum test for disease association with multiple common or rare variants. Hum Hered. 2010;70(1):42–54. 10.1159/000288704 20413981 PMC2912645

[8] Lippert C, Listgarten J, Liu Y, Kadie CM, Davidson RI, Heckerman D. FaST linear mixed models for genome-wide association studies. Nature methods. 2011;8(10):833–5. 10.1038/nmeth.1681 . 21892150

[9] Kang HM, Sul JH, Service SK, Zaitlen NA, Kong SY, Freimer NB, et al Variance component model to account for sample structure in genome-wide association studies. Nat Genet. 2010;42(4):348–54. 10.1038/ng.548 20208533 PMC3092069

[10] Zhang Z, Ersoz E, Lai CQ, Todhunter RJ, Tiwari HK, Gore MA, et al Mixed linear model approach adapted for genome-wide association studies. Nat Genet. 2010;42(4):355–60. 10.1038/ng.546 20208535 PMC2931336

[11] Svishcheva GR, Axenovich TI, Belonogova NM, van Duijn CM, Aulchenko YS. Rapid variance components-based method for whole-genome association analysis. Nat Genet. 2012;44(10):1166–70. 10.1038/ng.2410 . 22983301

[12] Neale BM, Rivas MA, Voight BF, Altshuler D, Devlin B, Orho-Melander M, et al Testing for an unusual distribution of rare variants. PLoS Genet. 2011;7(3):e1001322 10.1371/journal.pgen.1001322 21408211 PMC3048375

[13] Liu D, Lin X, Ghosh D. Semiparametric regression of multidimensional genetic pathway data: least-squares kernel machines and linear mixed models. Biometrics. 2007;63(4):1079–88. 10.1111/j.1541-0420.2007.00799.x 18078480 PMC2665800

[14] Liu D, Ghosh D, Lin X. Estimation and testing for the effect of a genetic pathway on a disease outcome using logistic kernel machine regression via logistic mixed models. BMC Bioinformatics. 2008;9:292 10.1186/1471-2105-9-292 18577223 PMC2483287

[15] Kwee LC, Liu D, Lin X, Ghosh D, Epstein MP. A powerful and flexible multilocus association test for quantitative traits. Am J Hum Genet. 2008;82(2):386–97. 10.1016/j.ajhg.2007.10.010 18252219 PMC2664991

[16] Wu MC, Kraft P, Epstein MP, Taylor DM, Chanock SJ, Hunter DJ, et al Powerful SNP-set analysis for case-control genome-wide association studies. Am J Hum Genet. 2010;86(6):929–42. 10.1016/j.ajhg.2010.05.002 20560208 PMC3032061

[17] Wu MC, Lee S, Cai T, Li Y, Boehnke M, Lin X. Rare-variant association testing for sequencing data with the sequence kernel association test. Am J Hum Genet. 2011;89(1):82–93. 10.1016/j.ajhg.2011.05.029 21737059 PMC3135811

[18] Li L, Zheng W, Lee JS, Zhang X, Ferguson J, Yan X, et al Collapsing-based and kernel-based single-gene analyses applied to Genetic Analysis Workshop 17 mini-exome data. BMC proceedings. 2011;5(Suppl 9 Genetic Analysis Workshop 17: Unraveling Human Exome DataS Ghosh, H Bickeboller, J Bailey, JE Bailey-Wilson, R Cantor, W Daw, AL DeStefano, CD Engelman, A Hinrichs, J Houwing-Duistermaat, IR Konig, J Kent Jr., N Pankratz, A Paterson, E Pugh, Y Sun, A Thomas, N Tintle, X Zhu, JW MacCluer and L Almasy):S117. 10.1186/1753-6561-5-S9-S117 22373309 PMC3287841

[19] Lee S, Wu MC, Lin X. Optimal tests for rare variant effects in sequencing association studies. Biostatistics. 2012;13(4):762–75. 10.1093/biostatistics/kxs014 22699862 PMC3440237

[20] Yang HC, Hsieh HY, Fann CS. Kernel-based association test. Genetics. 2008;179(2):1057–68. 10.1534/genetics.107.084616 18558654 PMC2429859

[21] Belonogova NM, Svishcheva GR, van Duijn CM, Aulchenko YS, Axenovich TI. Region-based association analysis of human quantitative traits in related individuals. PLoS One. 2013;8(6):e65395 10.1371/journal.pone.0065395 23799013 PMC3684601

[22] Schifano ED, Epstein MP, Bielak LF, Jhun MA, Kardia SL, Peyser PA, et al SNP set association analysis for familial data. Genet Epidemiol. 2012;36(8):797–810. 10.1002/gepi.21676 22968922 PMC3683469

[23] Chen H, Meigs JB, Dupuis J. Sequence kernel association test for quantitative traits in family samples. Genet Epidemiol. 2013;37(2):196–204. 10.1002/gepi.21703 23280576 PMC3642218

[24] Oualkacha K, Dastani Z, Li R, Cingolani PE, Spector TD, Hammond CJ, et al Adjusted sequence kernel association test for rare variants controlling for cryptic and family relatedness. Genet Epidemiol. 2013;37(4):366–76. 10.1002/gepi.21725 . 23529756

[25] Svishcheva GR, Belonogova NM, Axenovich TI. FFBSKAT: fast family-based sequence kernel association test. PLoS One. 2014;9(6):e99407 10.1371/journal.pone.0099407 24905468 PMC4048315

[26] Jiang D, McPeek MS. Robust rare variant association testing for quantitative traits in samples with related individuals. Genet Epidemiol. 2014;38(1):10–20. 10.1002/gepi.21775 . 24248908 PMC4510991

[27] Fan R, Wang Y, Mills JL, Wilson AF, Bailey-Wilson JE, Xiong M. Functional linear models for association analysis of quantitative traits. Genet Epidemiol. 2013;37(7):726–42. 10.1002/gepi.21757 24130119 PMC4163942

[28] Luo L, Zhu Y, Xiong M. Quantitative trait locus analysis for next-generation sequencing with the functional linear models. J Med Genet. 2012;49(8):513–24. 10.1136/jmedgenet-2012-100798 22889854 PMC3532851

[29] Luo L, Boerwinkle E, Xiong M. Association studies for next-generation sequencing. Genome Res. 2011;21(7):1099–108. 10.1101/gr.115998.110 21521787 PMC3129252

[30] Fan R, Wang Y, Mills JL, Carter TC, Lobach I, Wilson AF, et al Generalized functional linear models for gene-based case-control association studies. Genet Epidemiol. 2014;38(7):622–37. 10.1002/gepi.21840 25203683 PMC4189986

[31] Vsevolozhskaya OA, Zaykin DV, Greenwood MC, Wei C, Lu Q. Functional analysis of variance for association studies. PLoS One. 2014;9(9):e105074 10.1371/journal.pone.0105074 25244256 PMC4171465

[32] Ramsay J, Silverman BW. Functional Data Analysis: Springer; 2005. 430 p.

[33] Ramsay J, Hooker G, Graves S. Functional Data Analysis with R and MATLAB: Springer; 2009. 214 p.

[34] Horváth L, Kokoszka P. Inference for Functional Data with Applications: Springer; 2012. 422 p.

[35] Almasy L, Dyer TD, Peralta JM, Kent JW Jr, Charlesworth JC, Curran JE, et al Genetic Analysis Workshop 17 mini-exome simulation. BMC proceedings. 2011;5 Suppl 9:S2 10.1186/1753-6561-5-S9-S2 22373155 PMC3287854

[36] Weisberg S. Applied Linear Regression: Wiley; 2013. 368 p.

