# Supplementary figures and images for: Region-Based Association Test for Familial Data under Functional Linear Models
Source: PLoS One. 2015 Jun 25;10(6):e0128999. doi: 10.1371/journal.pone.0128999 (PMC4481467; doi:10.1371/journal.pone.0128999)

**(c) Causal 20%, unidirected 100%**

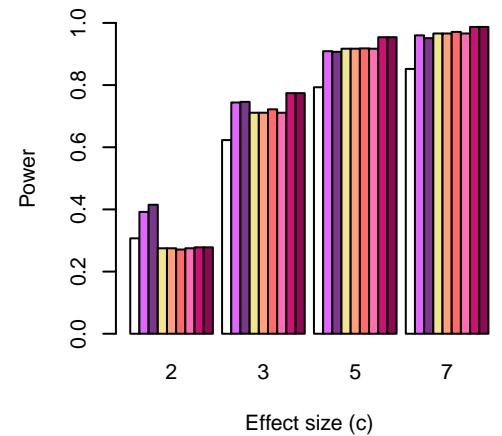

**(f) Causal 20%, unidirected 80%**

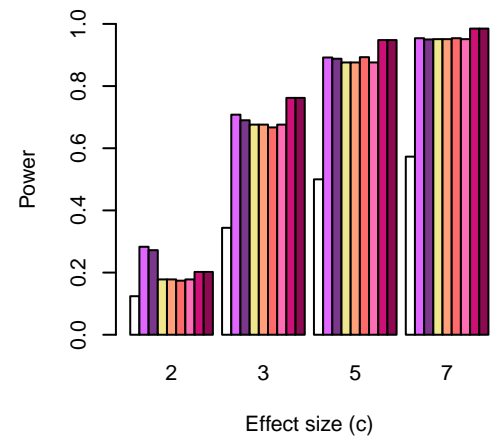

**(i) Causal 20%, unidirected 50%**

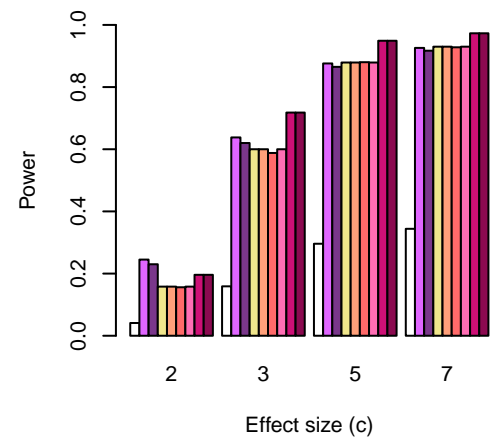

Supplement: S2 Fig — The notations of the methods are the same as in Fig 1. (PDF) [file pone.0128999.s002.pdf]
