# Supplementary material for: Region-Based Association Test for Familial Data under Functional Linear Models
Source: PLoS One. 2015 Jun 25;10(6):e0128999. doi: 10.1371/journal.pone.0128999 (PMC4481467; doi:10.1371/journal.pone.0128999)
Supplement: S3 Fig — The notations of the methods are the same as in Fig 1. (PDF) [file pone.0128999.s003.pdf]

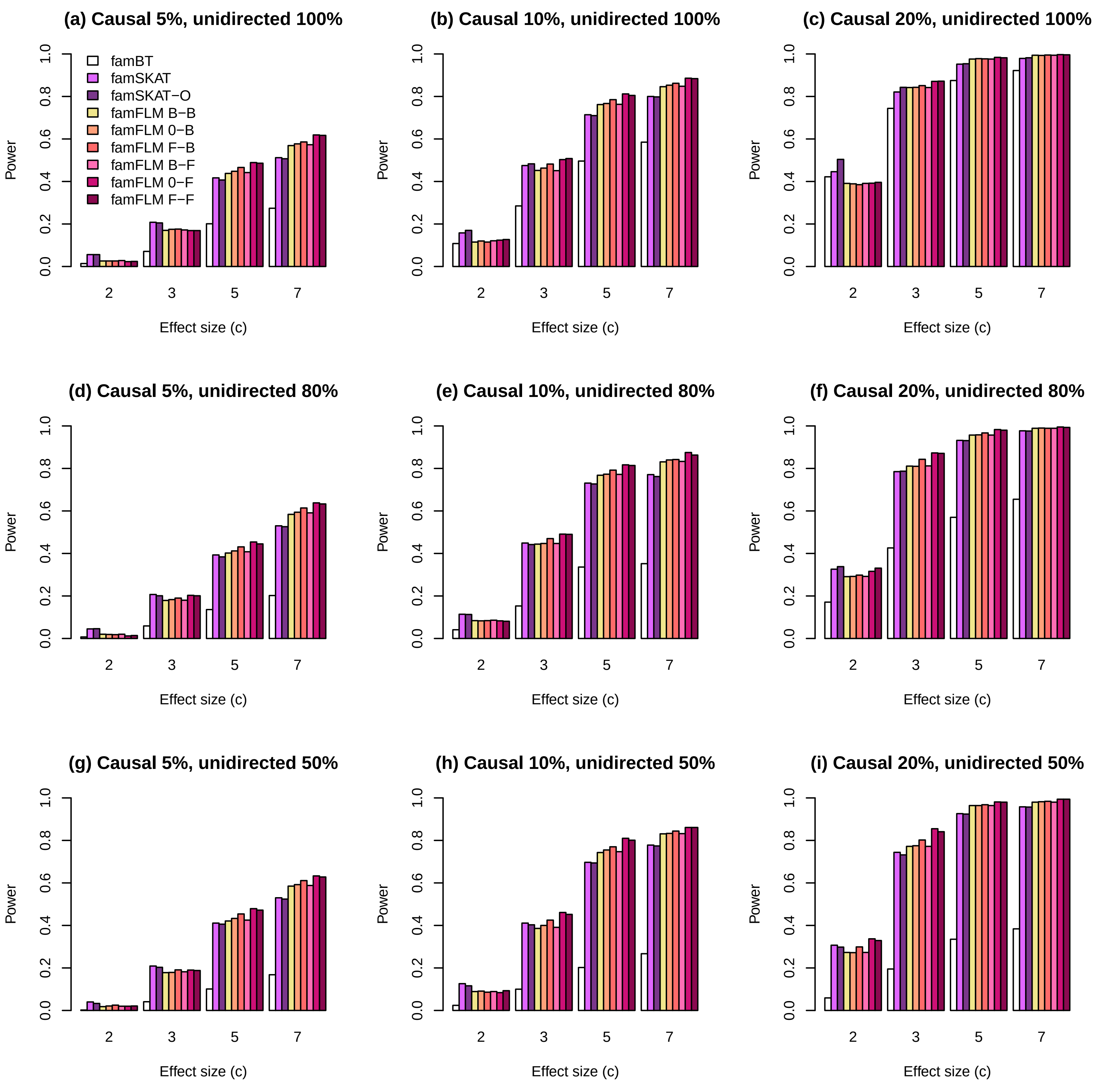

(a) Causal 5%, unidirected 100%
(b) Causal 10%, unidirected 100%
(c) Causal 20%, unidirected 100%
(d) Causal 5%, unidirected 80%
(e) Causal 10%, unidirected 80%
(f) Causal 20%, unidirected 80%
(g) Causal 5%, unidirected 50%
(h) Causal 10%, unidirected 50%
(i) Causal 20%, unidirected 50%
famBT
famSKAT
famSKAT−O
famFLM B−B
famFLM 0−B
famFLM F−B
famFLM B−F
famFLM 0−F
famFLM F−F
Power
0.0
0.2
0.4
0.6
0.8
1.0
2
3
5
7
Effect size (c)
