# Supplementary material for: Region-Based Association Test for Familial Data under Functional Linear Models
Source: PLoS One. 2015 Jun 25;10(6):e0128999. doi: 10.1371/journal.pone.0128999 (PMC4481467; doi:10.1371/journal.pone.0128999)
Supplement: S1 Note — (PDF) [file pone.0128999.s005.pdf]

S1 Note. Basis functions.

Contents

Types of basis functions 1

The Fourier basis 2

B-spline basis 3

Power and type I error rates with different numbers of basis functions 4

Table S1. Simulation results of type I error rates for famFLM test using cubic B-spline basis with different numbers of basis functions (K). 5

Table S2. Simulation results of type I error rates for famFLM test using Fourier basis with different numbers of basis functions (K). 5

Figure S1. The statistical power of regional association analysis on the familial data using cubic B-spline basis (a-c) or Fourier basis (d-f) with different numbers of basis functions (K). 6

References 7

Types of basis functions

A basis function system is a set of K standard mathematical functions, denoted by $\{\phi_1(t), \ldots, \phi_K(t)\}$. They are linearly independent and can be combined to estimate any function, denoted as $x(t)$. In this work, we estimated two specific functions $x(t) = \tilde{\beta}(t)$ and $x(t) = \tilde{G}(t)$, where $\tilde{\beta}(t)$ is the beta-smoothing function (BSF) and $\tilde{G}(t)$ is the genetic variant function (GVF), both from the functional linear model (2). There are several different types of basis functions that are selected, taking into account the behavior of the data. We considered two popular types of basis functions: *B-spline* and *Fourier bases.* The first type is more suitable for non-periodic data with open-ended range, and the second one is more suitable for the data with periodic or near-periodic nature with limited range.

Knowing only the values $\{x(t_i), i = 1,\ldots,m\}$ of an unknown function $x(t)$ in discrete points $\{t_i, i = 1,\ldots,m\}$, one can approximate $x(t)$ by a weighted sum (linear combination) of the basis functions:

$$x(t) \approx \sum_{k=1}^{K} c_k \phi_k(t) = c^T \phi(t),$$

where c is a $(K \times 1)$ vector of weight coefficients, and $\phi(t) = (\phi_1(t), \ldots, \phi_K(t))^T$. This approximation could also be carried out when the values $\{x(t_i), i = 1,\ldots,m\}$ are not given, but can been estimated.

For the functions $\tilde{\beta}(t)$ and $\tilde{G}(t)$, the approaches to finding the weight coefficients c differ. For estimating the GVFs in Model (2), a discrete realization of which is the known matrix $G(n \times m)$, we use a simple linear smoother that determines c^T as $G\Phi(\Phi^T\Phi)^{-1}$, where Φ $(m \times K)$ is the matrix with an (j,k)-th element equal to $\phi_k(t_j)$ [Ramsay and Silverman, 2005]. However, for estimating the BSF with unknown discrete realizations in Model (2), we find the vector c as unknown model parameters in regression linear equation.

When the values of $x(t)$ in points $\{t_i, i = 1, \ldots, m\}$ are given and the number of the basis functions K is equal to m, it is easy to see that such an exact solution of equation system $\{ x(t_i) = c^T \phi(t_i), i = 1, \ldots, m\}$ with regard to c exists. In this case, it doesn't matter what basis function system was used. However, when m is large, it is impractical to set K equal to m. When $K<m$, an accuracy of the approximated representation depends on selected type of basis function system.

Ideally, basis functions should have features that match with the known features of the function being estimated. It is easier to achieve a satisfactory approximation using a comparatively small number K of basis functions. We will consider in detail the *Fourier* and the *B-spline bases.*

The Fourier basis

The Fourier basis is a set of sine and cosine functions of increasing frequency, which is provided by the Fourier series:

$\varphi_0(t) = 1$,

$\varphi_{2r-1}(t) = \sin(2\pi rt)$ and $\varphi_{2r}(t) = \cos(2\pi rt)$, for $r = 1,\ldots,(K-1)/2$.

Here K is taken as a positive odd integer. Each function in this Fourier basis is periodic in t with period 1. If the discrete values of t_j are equally spaced on the normalized interval [0, 1], then this basis is orthogonal in the sense that the cross product matrix $\Phi^T\Phi$ is diagonal.

For a genome region with the m genetic variants, where $m \geq 25$, we, as Fan et al. [2013], selected $K = 25$.

Specifics: Fourier basis functions have excellent computational properties, especially if the discrete points of observation are equally spaced, due to the easy derivative estimation, and

due to the simple non-recursive construction technique. A Fourier series is especially useful for extremely stable functions, such as functions without strong local features where the curvature tends to be of the same order everywhere. However they are inappropriate for data where discontinuities in the function itself or in low order derivatives are known or suspected.

They are best suitable for describing data which are periodic or near-periodic. However, their periodicity is a problem for non-periodic data. See details in [e.g., Ramsay and Silverman, 2005; Ramsay et al., 2009; Ferraty and Romain, 2011; Horvath and Kokoszka, 2012].

B-spline basis

A B-spline basis is the most popular approximation system for non-periodic data. Here, a B-spline basis is a system of K polynomials of specified order d each (here, the order of a polynomial is the number of constants required to define it [Ramsay and Silverman, 2005; Ramsay et al., 2009]). An approximating function $x(t)$ is defined piecewise by basis polynomials with the given order of smoothness at the join points.

To use a B-spline basis, the interval normalized as [0, 1] is subdivided into L arbitrary segments, ($L = K – d + 1$). Consecutive segments are separated by a join point called a knot. The number of such interior points is equal to L–1. For each of consecutive segments, the approximating function $x(t)$ is defined as a corresponding basis polynomial. To make the resulting piecewise polynomial smoothing, the values of the polynomials and all their derivatives up to order d–2 must match at the join point for any pair of consecutive segments.

The i-th B-spline basis function of the k-th order ($k \le d$), defined on the set of all reals, and denoted by $B_{i,k}(t)$, $i = 1, \ldots, L+k-1$, can be defined recursively as follows:

$$B_{i,1}(t) = \begin{cases} 1, \mathit{if}\ t_i \le t < t_{i+1} \\ 0, \quad otherwize \end{cases} \quad \text{and}$$

$$B_{i,k}(t) = \frac{t - t_i}{t_{i+k-1} - t_i} B_{i,k-1}(t) + \frac{t_{i+k} - t}{t_{i+k} - t_{i+1}} B_{i+1,k-1}(t),\ k=2, \ldots, d.$$

Here $B_{i,k}(t)$ is a polynomial of order k that will be used on the i-th interval $t_i \le t < t_{i+1}$, i=1, …, L. Value k must be at least 2 and at most $L+1$. For each k, the resulting piecewise polynomial approximation in terms of $B_{i,k}$'s must have continuous derivatives up to order k–2 at all the knots.

For a genome region with the number of genetic variants $m \ge 15$, we selected $K = 15$ and $d = 4$, as Fan et al. [2013]. In this case, the corresponding number of knots is calculated as L –

$1 = K - d = 11$, the corresponding number of segments is 12, and the corresponding number of control points is 13.

Specifics: We used the cubic B-splines as a highest computationally feasible option. In this case, the running time is only slightly higher than in case of Fourier basis function. However, when the order of B-spline polynomials is high, the recursive construction technique can decelerate the calculating process. In addition, in a neighborhood of a knot that is distant from its neighboring knots, such splines could oscillate and deviate noticeably from the given approximating function. They can reduce the power of the methods. To use the B-spline basis we must determine not only the number of basis functions and the order of the polynomial segments but the location of knots. For computational convenience, we used equally spaced knots to determine B-spline basis. The power of the method can be increased, if the least squares fitting criterion to estimate location of knots on the base of analyzed data is used (Vsevolozhskaya et al., 2014). However this criterion is highly nonlinear in knot locations, and the computational challenges are severe. Nevertheless, in certain cases where strong curvature is localized in regions not known in advance, this is the more natural approach. The details can be found in [Ramsay and Silverman, 2005; Ramsay et al., 2009].

Power and type I error rates with different numbers of basis functions

We compared the statistical properties of our method using different number of basis functions (K) in a range 5–35. Two models, B-B and F-F, were selected for this testing:

- the model using Fourier basis for both BSF and GVF;
- the model using B-spline basis for both BSF and GVF.

The empirical type I error rates were very close to the declared values for all numbers of basis functions, both models and all tested scenarios (Tables S1-S2). Dependence of power on the number of basis functions varied for different scenarios (Fig S1). For scenarios with low genetic effect where power was ≤ 0.25 we did not see the difference between cases with different numbers of basis functions. For scenarios with middle and large genetic effect the worst result was obtained in case of 5 basis functions while other numbers of basis functions demonstrated about the same power. These results are in good agreement with the findings of Fan et al [27] that the statistical properties of the method do not strongly depend on the number of basis functions in a range of $10 \leq K \leq 25$. Therefore, we selected 15 and 25 basis function for B-spline and Fourier bases, respectively, as it was recommended by Fan et al [27].

With the number of basis functions in a range 15–35, the powers for models using Fourier basis were consistently higher than for corresponding models using B-spline basis (P values ≤ 0.006 in the paired t-tests).

Table S1. Simulation results of type I error rates for famFLM test using cubic B-spline basis with different numbers of basis functions (*K*).

α	**Numbers of basis functions (*K*)**			
	**5**	**15**	**25**	**35**
**0.05**	0.050649	0.050315	0.050264	0.050226
**0.01**	0.010223	0.010165	0.010167	0.010164
**0.001**	0.001035	0.001057	0.001043	0.001045
**0.0001**	0.000109	0.000110	0.000109	0.000108

Table S2. Simulation results of type I error rates for famFLM test using Fourier basis with different numbers of basis functions (*K*).

α	**Numbers of basis functions (*K*)**			
	**5**	**15**	**25**	**35**
**0.05**	0.050541	0.048556	0.047493	0.047083
**0.01**	0.010301	0.009851	0.009573	0.009530
**0.001**	0.001096	0.001037	0.001012	0.001006
**0.0001**	0.000107	0.000100	0.000102	0.000099

Figure S1. The statistical power of regional association analysis on the familial data using cubic B-spline basis (a-c) or Fourier basis (d-f) with different numbers of basis functions (*K*). All (rare and common) variants were used in simulations for selection of causal variants and in analysis. The proportion of causal variants having the same direction was 80%.

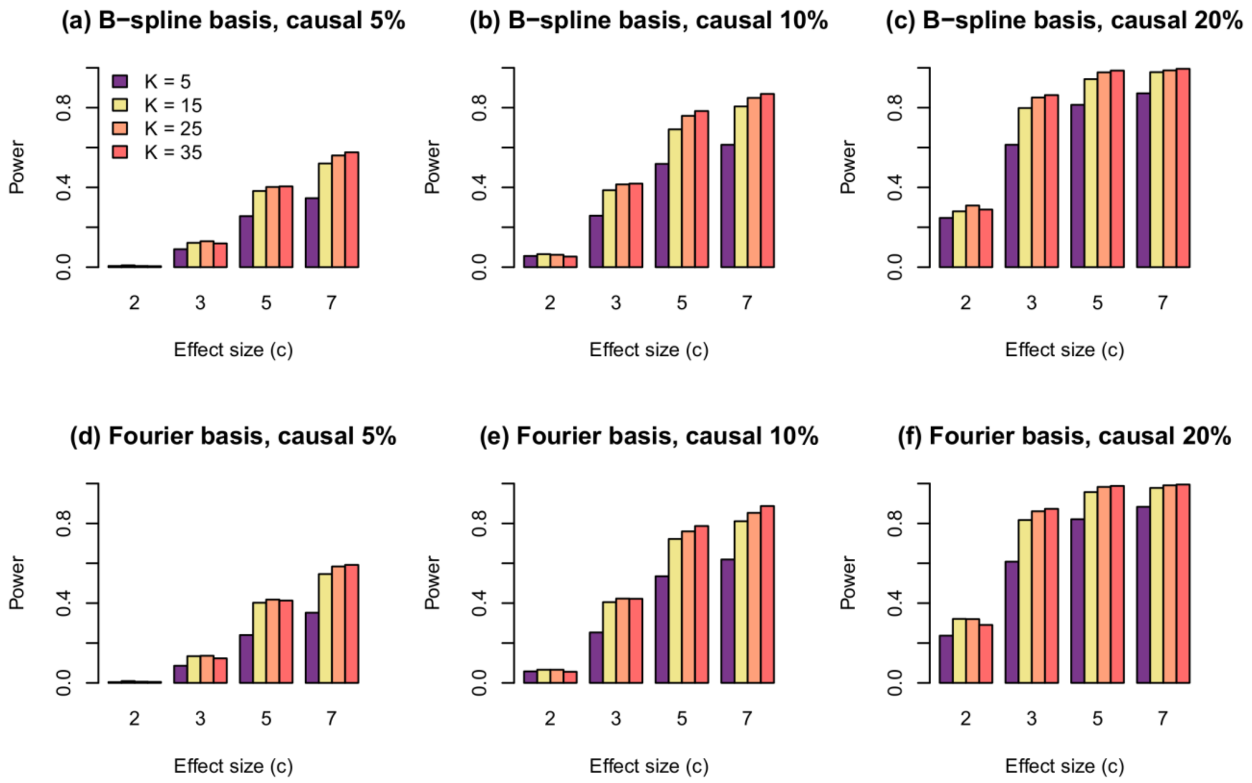

References

Fan R, Wang Y, Mills JL, Wilson AF, Bailey-Wilson JE, et al. (2013) Functional linear models for association analysis of quantitative traits. Genet Epidemiol 37: 726–742.

Ferraty F, Romain Y (2011) The Oxford Handbook of Functional Data Analysis (Eds), Oxford University Press, New York, NY, USA

Horvath L, Kokoszka P (2012) Inference for Functional Data with Applications. New York: Springer Series in Statistics. 422 p.

Ramsay JO, Hooker G, Graves S (2009) Functional Data Analysis with R and Matlab. New York: Springer-Verlag. 214 p.

Ramsay JO, Silverman BW (2005) Functional Data Analysis. New York: Springer Series in Statistics. 430 p.

Vsevolozhskaya OA, Zaykin DV, Greenwood MC, Wei C, Lu Q (2014) Functional analysis of variance for association studies. PLoS One. 22; 9(9):e105074. doi: 10.1371/journal.pone.0105074.
