# Supplementary material for: Region-Based Association Test for Familial Data under Functional Linear Models
Source: PLoS One. 2015 Jun 25;10(6):e0128999. doi: 10.1371/journal.pone.0128999 (PMC4481467; doi:10.1371/journal.pone.0128999)
Supplement: S2 Note — (PDF) [file pone.0128999.s006.pdf]

S2 Note. FLM-based association analysis under different ratios between *m*, K_G and K_β

Necessary conditions for performance of FLM-based method

In the framework of a functional linear model (FLM), we fix the number of basis functions (K_G to estimate genetic variant functions (GVF) and K_β to estimate beta-smooth function (BSF)) depending on the function basis type which can be different for GVF and BSF.

To perform association analysis using Model (5), we must estimate unknown betas, β_F, solving the equation

$$\beta_F = (W^T G^T \Omega^{-1} G W)^{-1} W^T G^T \Omega^{-1} (y - X\alpha), \tag{S1}$$

where matrices Ω, *G*, and *W* have dimensions $n \times n$, $n \times m$ and $m \times K_\beta$, respectively. Inverting the $K_\beta \times K_\beta$ square matrix $W^T G^T \Omega^{-1} G W$ in formula (S1) is possible if $n \geq m \geq K_\beta$. Therefore to unambiguously evaluate β_F, we must restrict the number of basis functions, K_β, as

$$K_\beta \leq m.$$

To introduce additional, stricter restrictions preventing an over-parameterization in Model (5), we present a matrix *W* of dimension $m \times K_\beta$ as a product of two matrices W_1 and W_2:

$$W_1 = \Phi(\Phi^T \Phi)^{-1} \tag{S2}$$

of dimension $m \times K_G$, and

$$W_2 = \int_0^1 \phi(t) \psi^T(t) dt \tag{S3}$$

of dimension $K_G \times K_\beta$.

Then the matrix $W^T G^T \Omega^{-1} G W$ in (S1) is transformed into ${W_2}^T {W_1}^T G^T \Omega^{-1} G W_1 W_2$. The additional restrictions must be imposed on dimensions of the matrices W_1 and W_2. Inverting the $K_\beta \times K_\beta$ square matrix ${W_2}^T {W_1}^T G^T \Omega^{-1} G W_1 W_2$ is possible if $n \geq m \geq K_G \geq K_\beta$. As a result, we must firstly restrict the number of basis functions for smoothing the genotypes, K_G, and then for smoothing the betas, K_β:

$$K_G \leq m \text{ and } K_\beta \leq K_G,$$

respectively. Therefore, if the declared number of the basis functions, K_G, used for GVF is more than *m*, K_G has to be reduced to *m*. Next, if the declared number of basis functions, K_β, used for BSF is more than K_G, K_β has to be reduced to K_G.

Equivalence of several functional linear models

In general, the functional linear models under consideration are described as

$$y = X\alpha + GW\beta_F + h + e, \tag{S4}$$

and an F-test statistic for them is calculated as

$$F = \frac{(RSS_0 - RSS_1)/K_\beta}{RSS_1/(n - K_\beta - 1)}.$$

Here $RSS_0 = (y - X\alpha)^T \Omega^{-1}(y - X\alpha)$, $RSS_1 = (y - X\alpha)^T P^T \Omega^{-1} P(y - X\alpha)$, and $P = I - GW(W^T G^T \Omega^{-1} GW)^{-1} W^T G^T \Omega^{-1}$.

The difference between the models with and without the smoothing on the GVF is in the way the matrix W is constructed. For all models using both the GVF and the BSF (i.e., F-F, B-B, F-B and B-F models), the matrix W is formed as product of two matrices W_1 and W_2, defined by expressions (S2) and (S3). The matrix W_1 of dimension $m \times K_G$ participates in the genotypes smoothing on base of one function basis, and the matrix W_2 of dimension $K_G \times K_\beta$ participates in the betas smoothing (under condition of the GVF smoothing), using two function bases. For the models which do not use the GVF (i.e., 0-F and 0-B models), the matrix W of dimension $m \times K_\beta$ is intended to smooth only the betas on the base of only one function basis and constructed analogously to the $m \times K_G$ matrix Φ in Model (5).

For the models with both the GVF and BSF, the matrix P looks as:

$$P = I - GW_1W_2(W_2^T W_1^T G^T \Omega^{-1} GW_1W_2)^{-1} W_2^T W_1^T G^T \Omega^{-1}. \tag{S5}$$

If $m > K_G$ and $K_G = K_\beta$, the matrix W_2 is invertible, and hence it can be canceled in the expression for P (S5). Moreover, decomposing W_1 into Φ and $(\Phi^T\Phi)^{-1}$ (see formula (S2)) allows us also to cancel the matrix $(\Phi^T\Phi)^{-1}$ in the expression for P (S5). As a result, the matrix W is reduced to the matrix Φ. Therefore, the models within groups (0-F, F-F, and F-B) and (0-B, B-B and B-F) do not differ from each other because the matrix Φ for the models with the GVF and the matrix W for the models without the GVF are identical.

If $m \geq K_G$ and $K_G > K_\beta$, the matrix W_2 is not invertible (because it is not square) and it cannot be canceled in the expression for P (S5). As a result, all the models are different because matrices W in the models with both the GVF and the BSF and the models with only the GVF are constructed differently.

In our study, we fixed the number of basis functions as 25 for Fourier series and as 15 for B-splines. Therefore:

- The B-B model is equivalent to the 0-B model, and the F-F model is equivalent to the 0-F model.
- The F-B model is equivalent to none of the used models.
- In the B-F model, K_G (originally declared as 25) is automatically reduced to 15 to meet condition $K_G \geq K_\beta$. This model is equivalent to the models 0-B and B-B.

In summary, the set of all six models used in our study is reduced to the set of three models being different from each other: 0-B (identical to B-B and B-F), 0-F (identical to F-F) and F-B.

Analysis of regions with small number of genetic variants

For the situations, when the number of genetic variants is small (smaller than the number of basis functions), to avoid model over-parameterization, we imposed restrictions on the number of basis functions, reducing K_G and K_β to the number of variants in a region of interest. As a result, if K_G and K_β become equal to m, the functional linear model (5), described by the equation (S4), is reduced to a more simple linear mixed model (1), described by the equation

$$y = X\alpha + G\beta + h + e,$$

because the matrix W becomes invertible and it can be canceled in the expression for P (S5). Therefore, the benefit of the proposed method compared to simple approach based on linear mixed model exists only for regions with the large number of variants.
