# Supplementary material for: Region-Based Association Test for Familial Data under Functional Linear Models
Source: PLoS One. 2015 Jun 25;10(6):e0128999. doi: 10.1371/journal.pone.0128999 (PMC4481467; doi:10.1371/journal.pone.0128999)

S3 Note. Effect of the sample size and the number of genetic variants on the running time of famFLM test.

To explore the dependence between the running time (RT) and both the sample size (n) and the number of genetic markers in the region (m), we analyzed regions of fixed length (m = 30, 50, 70, and 100) and sample sizes n = 500, 1000, 2000, and 4000.

We divided the total running time into three components:

- The running time of the maximum likelihood estimation of parameters under the null model (RT_0). It depends strongly on sample size, but needs to be run once for a trait.
- The running time of common for all regions transformations of phenotypes and calculations of the functional basis (RT_1). It depends mainly on the sample size and slightly on the type of functional model. This step is performed once for each exome analysis.
- The running time of the analysis of a region itself where genotypes are processed (RT_2). The duration of this step depends on both n and m.

For fixed m, the total running time of exome analysis can be therefore represented as

$$RT = RT_0 + RT_1 + (\text{number of regions})*RT_2.$$

We estimated RT_0, RT_1, and RT_2 for different sample sizes and region sizes. Results for six models of famFLM are presented in Table S1. All the models showed about the same performance with $RT_0 > RT_1 > RT_2$. Hence, if only one region is analyzed, the RT is determined primarily by RT_0 and RT_1, but not by RT_2. However, for analysis of a whole exome with thousands of regions, the duration of regional analysis itself (sum of RT_2 by regions) would many times exceed the value of ($RT_0 + RT_1$). In such a large study, RT_2 would become critical.

Dependence of RT_2 on sample size is analytically quadratic. However, in our experiment the best predictor was $n^{1.5}$ (e.g. $RT_2 = 1.86 \times 10^{-6} \times n^{1.55}$, $R^2 = 0.985$, $P = 4.94 \times 10^{-3}$ for famFLM 0-F and $m = 50$). This probably can be due to some algorithmic properties of matrix processing. Figure S1 shows the running time (RT_2) of the FDA-based and the kernel-based methods plotted against the sample size. Methods have similar performance for all tested sample sizes. Therefore, we conclude that our FDA-based method is expected to be at least as fast as the kernel-based regional association methods even for large studies where the running time is a major concern.

Figure S2 shows that the values of RT_2 increase linearly with m (e.g. $RT_2 = 0.064 + 0.014 \times m$, $R^2 = 0.999$, $P = 3.4 \times 10^{-4}$ for famFLM 0-F and $n = 4000$).

Table S1. The running time of FDA-based regional association analyses for different sample sizes (*n*) and region sizes (*m*).

		m	**30**				**50**			
		n	**500**	**1000**	**2000**	**4000**	**500**	**1000**	**2000**	**4000**
RT[*]	**Model**									
RT_0	famFLM B-B[**]		0.64	4.09	33.70	272.52	0.64	4.09	43.72	272.57
	famFLM 0-B		0.64	4.11	33.97	272.50	0.64	4.09	34.57	272.49
	famFLM F-B		0.64	4.17	33.96	272.57	0.64	4.11	48.68	272.51
	famFLM B-F		0.64	4.09	33.89	272.63	0.64	4.10	40.78	272.59
	famFLM 0-F		0.64	4.11	33.95	272.61	0.64	4.08	43.62	272.53
	famFLM F-F		0.64	4.08	33.90	272.60	0.64	4.09	48.04	272.58
RT_1	famFLM B-B		0.13	0.86	7.54	60.50	0.13	0.86	10.86	60.51
	famFLM 0-B		0.11	0.84	7.45	60.47	0.11	0.84	7.66	60.49
	famFLM F-B		0.13	0.86	7.49	60.48	0.12	0.86	8.51	60.49
	famFLM B-F		0.13	0.86	7.55	60.50	0.13	0.86	10.47	60.50
	famFLM 0-F		0.11	0.84	7.47	60.47	0.11	0.84	8.44	60.47
	famFLM F-F		0.12	0.85	7.47	60.48	0.12	0.86	8.55	60.49
RT_2	famFLM B-B		0.02	0.05	0.14	0.49	0.03	0.07	0.30	0.75
	famFLM 0-B		0.02	0.05	0.14	0.48	0.03	0.07	0.22	0.74
	famFLM F-B		0.02	0.05	0.14	0.49	0.03	0.07	0.22	0.75
	famFLM B-F		0.03	0.05	0.14	0.49	0.03	0.07	0.28	0.75
	famFLM 0-F		0.02	0.05	0.14	0.49	0.03	0.07	0.24	0.75
	famFLM F-F		0.03	0.05	0.14	0.49	0.03	0.07	0.27	0.76

Table S1. Cont.

		m	70				100			
		n	500	1000	2000	4000	500	1000	2000	4000
RT [*]	**Model**									
RT_0	famFLM B-B[**]		0.63	4.19	33.75	272.61	0.63	4.17	33.89	272.92
	famFLM 0-B		0.63	4.10	33.78	272.50	0.63	4.04	33.78	273.46
	famFLM F-B		0.63	4.16	33.85	272.39	0.63	4.10	33.87	272.34
	famFLM B-F		0.63	4.10	33.86	272.51	0.63	4.09	33.89	273.46
	famFLM 0-F		0.62	4.16	33.75	272.47	0.63	4.05	33.88	273.53
	famFLM F-F		0.63	4.20	33.64	272.41	0.63	4.17	33.90	272.25
RT_1	famFLM B-B		0.13	0.86	7.51	60.50	0.13	0.85	7.52	60.49
	famFLM 0-B		0.11	0.84	7.49	60.50	0.11	0.84	7.54	60.46
	famFLM F-B		0.13	0.85	7.50	60.50	0.12	0.85	7.52	60.49
	famFLM B-F		0.13	0.85	7.50	60.51	0.12	0.85	7.52	60.49
	famFLM 0-F		0.11	0.84	7.43	60.51	0.11	0.83	7.44	60.39
	famFLM F-F		0.12	0.85	7.50	60.49	0.12	0.85	7.52	60.49
RT_2	famFLM B-B		0.04	0.09	0.28	1.02	0.05	0.12	0.40	1.45
	famFLM 0-B		0.04	0.08	0.28	1.02	0.05	0.12	0.44	1.53
	famFLM F-B		0.04	0.09	0.28	1.02	0.05	0.12	0.40	1.45
	famFLM B-F		0.04	0.09	0.28	1.03	0.05	0.12	0.40	1.46
	famFLM 0-F		0.04	0.09	0.28	1.02	0.05	0.12	0.40	1.46
	famFLM F-F		0.04	0.09	0.29	1.03	0.05	0.12	0.41	1.46

[*] See text for definitions of RT_1, RT_2, and RT_3.

[**] For famFLM, six functional models were tested: B-spline basis for both the GVF and the BSF (B-B), only the BSF described via B-spline basis (0-B), Fourier basis for the GVF and B-spline basis for the BSF (F-B), B-spline basis for the GVF and Fourier basis for the BSF (B-F), only the BSF described via Fourier basis (0-F), Fourier basis for both the GVF and the BSF (F-F).

Figure S1. The running time of regional association analyses (*RT_2*) for different sample sizes and different numbers of genetic variants in the region (*m*). For the FDA-based approach, only the model with BSF described via Fourier basis (0-F) is shown (other models demonstrate similar behavior as can be seen from Table S1). For kernel-based method (famSKAT), the 'FFBSKAT' function was used.

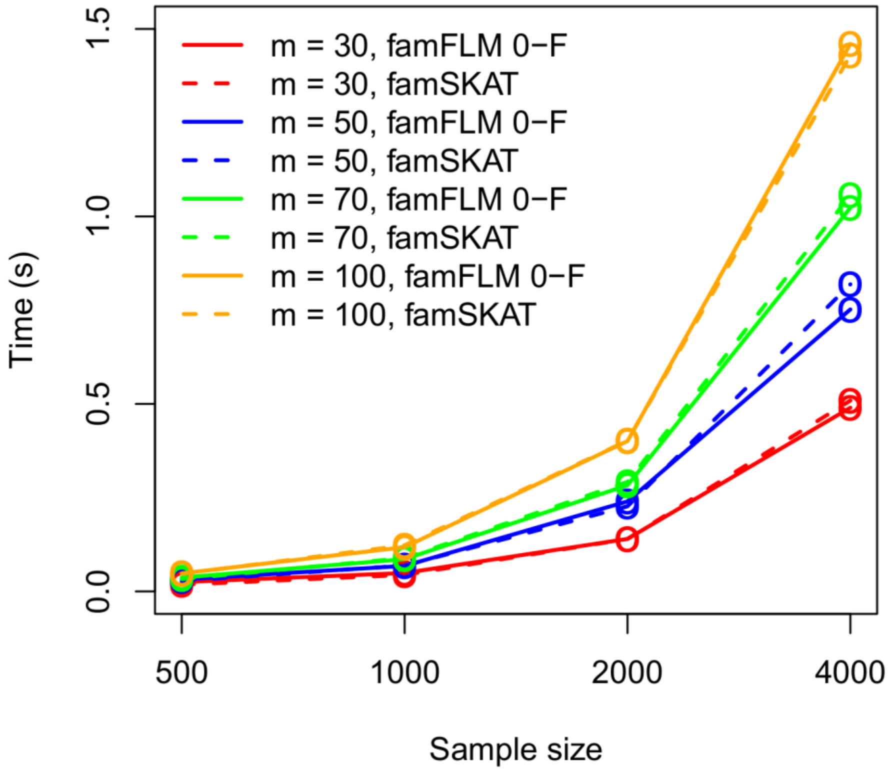

Figure S2. The running time of regional association analyses (*RT_2*) for different sample sizes (*n*) plotted against the number of genetic variants in the region. For the FDA-based approach, only the model with BSF described via Fourier basis (0-F) is shown (other models demonstrate similar behavior as can be seen from Table S1).

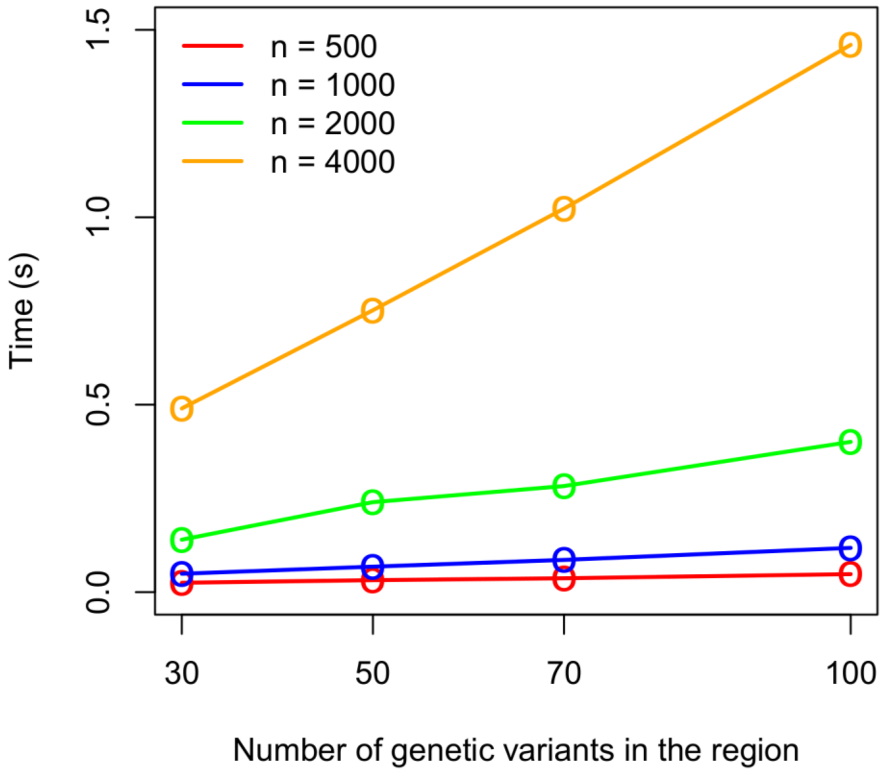

Supplement: S3 Note — (PDF) [file pone.0128999.s007.pdf]
